# Supplementary material for: Extracellularly Released Calreticulin Induced by Endoplasmic Reticulum Stress Impairs Syncytialization of Cytotrophoblast Model BeWo Cells
Source: Cells. 2021 May 24;10(6):1305. doi: 10.3390/cells10061305 (PMC8225044; doi:10.3390/cells10061305)
Supplement: Supplementary file 1 [file cells-10-01305-s001.zip › cells-1220445-supplementary.pdf]

Supplemental Materials

Extracellularly released calreticulin induced by endoplasmic reticulum stress impairs syncytialization of cytotrophoblast model BeWo cells

Naoyuki Iwahashi,[1] Midori Ikezaki,[2] Kazuchika Nishitsuji,[2,*] Madoka Yamamoto,[1] Ibu Matsuzaki,[3] Naoki Kato,[2] Naoyuki Takaoka,[2] Mana Taniguchi,[2] Shin-ichi Murata,[3] Kazuhiko Ino,[1] and Yoshito Ihara[2,*]

[1]Department of Obstetrics and Gynecology, Wakayama Medical University School of Medicine, 811-1 Kimiidera, Wakayama 641-8509, Japan; [2]Department of Biochemistry, Wakayama Medical University School of Medicine, 811-1 Kimiidera, Wakayama 641-8509, Japan; [3]Department of Human Pathology, Wakayama Medical University School of Medicine, 811-1 Kimiidera, Wakayama 641-8509, Japan

*Correspondence: Kazuchika Nishitsuji, Ph.D., Department of Biochemistry, Wakayama Medical University School of Medicine, 811-1 Kimiidera, Wakayama 641-8509, Japan, Phone/fax: +81-73-441-0628, E-mail address: nishit@wakayama-med.ac.jp; Yoshito Ihara, M.D., Ph.D., Department of Biochemistry, Wakayama Medical University School of Medicine, 811-1 Kimiidera, Wakayama 641-8509, Japan, Phone/fax: +81-73-441-0628, E-mail address: y-ihara@wakayama-med.ac.jp

Supplemental Procedures

Measurement of serum protein concentration

Protein concentrations of serum samples were determined by means of the Pearce BCA protein assay kit (Thermo Fisher Scientific, Waltham, MA, USA) according to the manufacture's protocol.

Supplemental Table S1 Protein concentrations of serum samples

Sample ID	Protein concentration (mg/mL)
No pregnancy #1	87.8
No pregnancy #2	82.5
No pregnancy #3	82.9
No pregnancy #4	81.8
No pregnancy #5	73.6
No pregnancy #6	90.4
Normal pregnancy #1	78.8
Normal pregnancy #2	84.7
Normal pregnancy #3	110.3
Normal pregnancy #4	102.4
Normal pregnancy #5	99.3
Normal pregnancy #6	92.9
PE #1	86.2
PE #2	96.7
PE #3	111.5
PE #4	78.7
PE #5	102.5
PE #6	112.7
